# YOLO11-FR: A bridge crack detection method based on frequency-domain fusion and an edge enhancement mechanism

**DOI:** 10.1371/journal.pone.0354254

**Published:** 2026-07-17

**Authors:** Yangming Zhang, Baohui Tian, Hufeng Guo

**Affiliations:** 1 Department of Traffic Information Engineering, Henan College of Transportation, Zhengzhou, China; 2 State Key Laboratory of Extreme Environment Optoelectronic Dynamic Measurement Technology and Instrument, Taiyuan, China; Hohai University, CHINA

## Abstract

Bridge cracks are important indicators of structural deterioration, and accurate crack detection is essential for bridge operation, maintenance, and safety assessment. However, crack detection remains challenging because cracks are often slender, low-contrast, and easily confused with concrete texture, stains, and other background patterns. To address these problems, this paper proposes YOLO11-FR, an improved YOLO11-based bridge crack detector that integrates a Fused Fourier Conv Mixer (FFCM) and a Residual Edge Enhancement Module (REEM). FFCM combines local convolution with a gated residual Fourier branch to introduce full-spectrum Fourier-domain feature interaction, while lightweight gating and bounded residual scaling regulate the reconstructed response. REEM enhances crack boundary features using local and dilated depthwise branches, horizontal and vertical stripe-convolution branches, a Sobel edge prior, and channel-spatial gates. Comparative experiments were conducted on GYU-DET-Crack, a crack subset extracted from the public GYU-DET dataset. Compared with the YOLO11n baseline, YOLO11-FR improves mAP50 and mAP50-95 by 3.4 and 3.7 percentage points, respectively. Validation on the Crack500 crack dataset further shows that YOLO11-FR increases mAP50 from 56.0% to 57.7% and mAP50-95 from 32.7% to 34.8%. These results indicate that the proposed YOLO11-FR improves bridge crack detection accuracy and provides a practical detection approach for crack screening under complex concrete surface backgrounds.

## 1. Introduction

As a core component of transportation infrastructure, the structural health of bridges is directly associated with traffic safety and regional traffic efficiency [1–3]. Cracks represent the most prevalent form of diseases in bridge concrete structures. The initiation and propagation of cracks will gradually diminish the bearing capacity and durability of the structure and might result in more severe structural safety risks [4,5]. In China, there is a substantial number of highway bridges, and they generally suffer from diseases such as cracks to varying degrees during their long-term service. Therefore, efficient and accurate crack detection technologies are urgently required to achieve early identification and early warning of diseases.

Bridge inspection is no longer limited to manual visual inspection. In current engineering practice, manual inspection is often combined with image-based inspection, UAV-assisted imaging, and sensor-based monitoring. Manual inspection remains useful for direct assessment, but it is labor intensive, subjective, and affected by lighting conditions, inspector experience, and accessibility of the bridge surface. Image-based and UAV-based methods improve inspection efficiency and safety, yet crack detection from images is still challenging because fine cracks can be weak, discontinuous, and embedded in complex concrete textures. Traditional image processing methods, including threshold segmentation [6], edge detection [7], texture analysis [8], and early handcrafted crack detection pipelines [9,10], rely heavily on manually designed operators and parameter settings; therefore, they are sensitive to noise, shadows, water stains, corrosion, spalling, and surface roughness.

Deep learning provides a more adaptive solution for crack detection. CNN-based semantic segmentation methods [11–16] can provide pixel-level crack masks, but their computational cost and annotation requirements may limit real-time deployment in engineering inspection. Object detectors such as Faster R-CNN and SSD [17,18] output crack locations with bounding boxes and are therefore attractive for rapid bridge crack screening. Among one-stage detectors, the YOLO series has been widely used in crack detection because of its end-to-end detection framework, fast inference speed, and favorable balance between accuracy and computational efficiency. YOLO11 [19] was selected as the direct baseline in this study because it inherits the real-time design of the YOLO family, uses a lightweight detection framework, and provides a suitable foundation for edge-oriented bridge crack screening. Nevertheless, standard spatial-domain convolution mainly aggregates information within local neighborhoods, while repeated downsampling may attenuate weak and spatially narrow crack responses. Concrete textures, stains, and surface irregularities can generate similar local activations, making true crack responses difficult to distinguish from background patterns and causing false detections or discontinuous localization along fine crack branches.

To address these limitations, this paper constructs YOLO11-FR for bridge crack detection. The proposed model introduces FFCM for local spatial processing and gated full-spectrum Fourier-domain refinement, and REEM for residual edge enhancement. In the modified architecture, FFCM is applied only to the high-level P5 branch to complement local spatial modeling through Fourier-domain component interaction, whereas REEM is applied to P3, P4, and P5 features to refine boundary-oriented responses at multiple scales. The Fourier branch is implemented as a bounded residual refinement rather than an explicit high-frequency selector, thereby preserving the original YOLO11 feature-fusion pathway and limiting disturbance to the baseline representation.

The main contributions of this study are summarized as follows. First, a residual FFCM module is introduced to combine local multi-scale convolution with a learned transformation of the complete Fourier representation; the reconstructed branch is regulated by lightweight gating and bounded residual scaling rather than explicit frequency-band selection. Second, a multi-branch REEM module is designed with local, dilated, horizontal and vertical stripe-convolution, and Sobel-prior branches to refine crack boundary responses. Third, the two modules are integrated into the YOLO11 detection head while preserving the original feature-fusion pathway, and the modified model is evaluated through module ablations, three-seed experiments, and independent training on a second public crack dataset without offline augmentation-based dataset expansion.

## 2. Related work

### 2.1. Bridge crack detection methods

Bridge crack detection research can be broadly divided into traditional image processing, semantic segmentation, and object detection. Traditional methods such as thresholding, edge detection, and texture analysis are computationally efficient, but they are sensitive to imaging conditions and often require manually tuned parameters. Segmentation-based deep networks can delineate crack pixels more precisely, but they require pixel-level annotations and usually introduce higher computational cost. Object detection methods provide a practical alternative for fast inspection because they localize cracks by bounding boxes and are easier to deploy in real-time screening systems. However, YOLO-based crack detectors still face two unresolved issues: fine crack edges can be weakened during feature extraction and downsampling, and complex concrete textures can be incorrectly activated as crack-like features.

Existing YOLO-based crack detection methods mainly improve the backbone and feature-fusion network [20–22], attention or deformation modules [23,24], and lightweight detection heads or deployment-oriented architectures [25–30]. Although these modifications can improve multi-scale representation and detection efficiency, fine crack boundaries may still be weakened during repeated feature extraction and downsampling. In addition, the elongated and directionally variable morphology of cracks requires more specialized feature modeling than that provided by general-purpose object detectors. Frequency-domain feature interaction and crack-oriented edge enhancement are therefore reviewed separately in the following sections.

### 2.2. YOLO11

**Fig 1:**
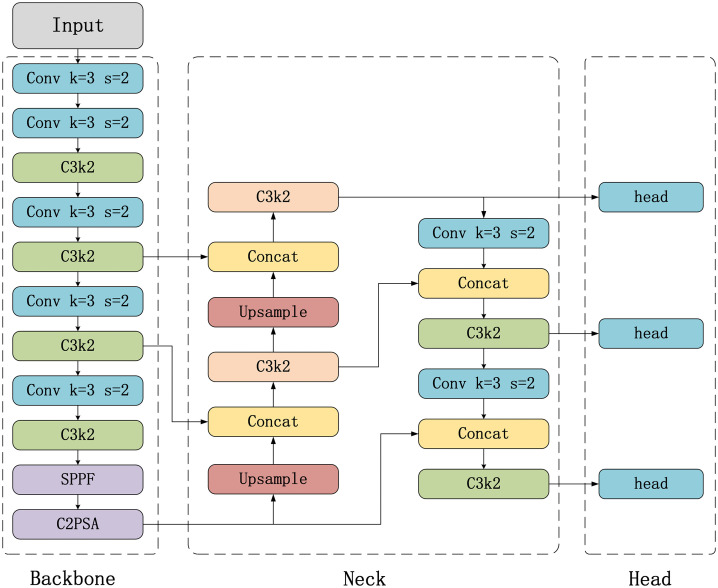
Baseline YOLO11 architecture used in this study.

YOLO11 is a lightweight detector in the YOLO series released by Ultralytics. It follows the end-to-end detection paradigm of the YOLO family and maintains real-time inference capability through a compact backbone, multi-scale feature fusion, and an efficient detection head. As shown in Fig 1, its architecture consists of a backbone for hierarchical feature extraction, a neck for fusing multi-scale features, and a detection head for predicting object locations and categories. This lightweight structure makes YOLO11 suitable as the baseline model in this study.

However, bridge cracks are typically slender, low-contrast, and discontinuous, and the standard YOLO11 architecture is not specifically designed to preserve weak crack responses after repeated feature extraction and downsampling. In addition, concrete textures, stains, and surface irregularities may produce crack-like edge responses, making true crack boundaries difficult to distinguish from background patterns. Therefore, this study builds upon YOLO11 by introducing residual full-spectrum Fourier-domain interaction to enhance its high-level feature representation and residual edge enhancement to refine multi-scale crack boundaries.

### 2.3. Frequency-domain feature modeling for crack detection

Frequency-domain transforms provide a complementary representation to spatial-domain convolution because each Fourier coefficient is computed from the entire spatial feature map. This property allows frequency-domain feature processing to introduce global feature interaction, whereas standard convolution mainly aggregates local neighborhood information. Slender cracks may produce rapid local intensity variations that contribute to relatively high-frequency components. However, concrete texture, stains, corrosion, shadows, and imaging noise can generate similar spectral responses. Therefore, high-frequency energy cannot be directly regarded as crack-specific information, and unconstrained high-frequency amplification may increase false responses.

Previous frequency-domain studies in image restoration, low-light enhancement, and small-object detection [31–33] suggest that spectral representations can complement local spatial convolution by improving global feature interaction and enhancing responses to weak or degraded structures. However, bridge crack detection differs from these tasks because crack-like background textures and surface interference may also produce strong frequency responses. These studies therefore motivate the use of frequency-domain interaction in this work, but not direct frequency amplification, fixed high-pass filtering, or predefined frequency-band selection.

Motivated by these frequency-aware methods, this study adapts FFCM as an auxiliary high-level refinement component for bridge crack detection. Instead of decomposing the input into predefined low- and high-frequency bands, FFCM operates on the complete feature spectrum. It applies a real-valued fast Fourier transform to the input feature, transforms the concatenated real and imaginary components, and reconstructs the Fourier-branch response through an inverse Fourier transform. The reconstructed response is regulated by lightweight gating and bounded residual scaling before being fused with the original YOLO feature representation.

Through this design, FFCM is interpreted as a gated full-spectrum Fourier-domain refinement module rather than a frequency-band enhancement module. Its purpose is to complement local spatial convolution with global frequency-domain interaction while limiting excessive disturbance to the original YOLO feature representation and reducing the risk of amplifying crack-like background textures. The detailed architecture and formulation of FFCM are presented in Section 3.1.

### 2.4. Edge enhancement and directional feature modeling for crack detection

Edge information is important for crack detection because cracks are commonly characterized by narrow intensity transitions, irregular boundaries, and elongated spatial structures. Traditional edge operators provide explicit gradient cues, but fixed filters are sensitive to concrete texture, stains, shadows, corrosion, and imaging noise [7,10]. Deep-learning-based crack detection methods therefore incorporate multi-scale convolution, attention mechanisms, and directional feature modeling to improve boundary representation [13,24,34]. Table 1 summarizes the main advantages and remaining limitations of these representative strategies.

**Table 1:** Summary of representative edge-enhancement strategies for crack detection.

Strategy	Main improvement	Remaining limitation
Fixed gradient operators	Provide explicit gradient cues.	Sensitive to texture, stains, shadows, corrosion, and noise.
Multi-scale or dilated convolution	Capture local patterns and wider context.	Limited modeling of elongated directional continuity.
Attention-based enhancement	Reweight channel or spatial responses adaptively.	Do not explicitly encode crack boundaries or morphology.
Directional feature modeling	Capture elongated responses along specific directions.	Limited directions cannot fully represent curved, branched, or discontinuous cracks.

As summarized in Table 1, no individual strategy fully addresses the joint requirements of bridge crack detection. Fixed gradients provide interpretable edge cues but can respond strongly to background texture; multi-scale convolution captures broader context but does not explicitly preserve elongated directional continuity; general-purpose attention reweights features without directly extracting crack boundaries; and directional modeling alone cannot represent all curved, branched, and discontinuous morphologies. Furthermore, directly adding edge-enhanced responses may disturb pretrained detector features if their contribution is not controlled.

These observations motivate a combined edge-refinement design. The proposed REEM integrates a local depthwise branch for fine boundary extraction, a dilated branch for wider contextual modeling, horizontal and vertical stripe-convolution branches for elongated directional structures, and a fixed Sobel-prior branch for explicit gradient guidance. Channel-spatial gates and bounded residual scaling regulate the fused response before it is added to the original feature. The detailed architecture and formulation are presented in Section 3.2.

## 3. Method

**Fig 2:**
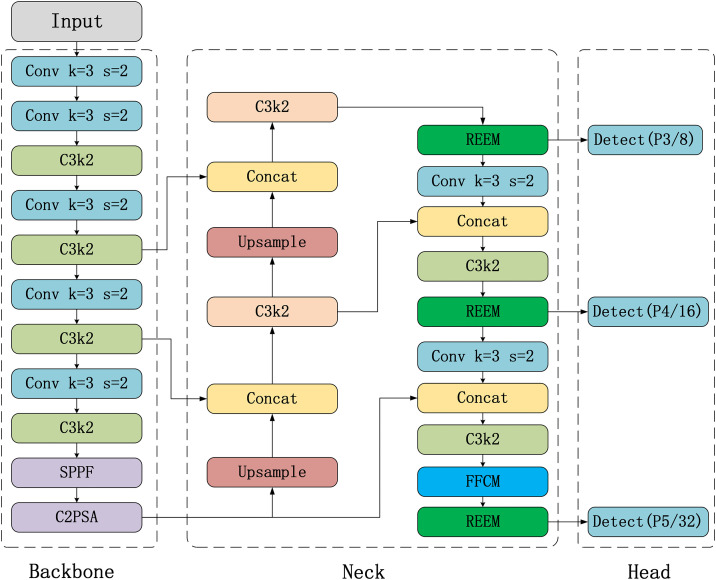
Overall architecture of YOLO11-FR.

YOLO11-FR is designed as a residual refinement framework based on the YOLO11 detection head. The original YOLO11 backbone and neck feature-fusion pathway are retained to preserve the baseline multi-scale representation and training stability. As shown in Fig 2, the proposed refinement modules are inserted into the detection head after the corresponding C3k2 blocks. Specifically, REEM is applied to the P3/8 and P4/16 detection branches to refine fine crack boundaries in relatively high-resolution features. On the P5/32 branch, FFCM is first used to perform residual frequency-domain and local spatial feature fusion, and REEM is then applied for final edge refinement. The refined P3/8, P4/16, and P5/32 features are fed into the Detect heads for crack localization.

This design separates the roles of the two modules. FFCM performs auxiliary high-level feature refinement by introducing full-spectrum Fourier-domain component interaction and gated residual fusion, thereby complementing standard spatial-domain convolution. REEM directly targets crack-oriented edge representation by combining local, dilated, directional stripe, and Sobel-prior branches. By integrating both modules in a residual manner, YOLO11-FR adaptively refines crack-related representations while limiting excessive disturbance to the original YOLO11 feature-fusion pathway.

### 3.1. Fused Fourier Conv Mixer

Inspired by recent frequency-domain feature modeling in image restoration [31], the Fused Fourier Conv Mixer (FFCM) is introduced to refine high-level detection features by combining local spatial perception with Fourier-domain feature interaction. In this study, FFCM is adapted as a residual high-level refinement component for bridge crack detection and inserted into the P5 detection branch of YOLO11. Its residual Fourier branch complements local convolutional features, while lightweight gating and bounded residual scaling regulate the contribution of the reconstructed Fourier-domain response to the original feature representation.

**Fig 3:**
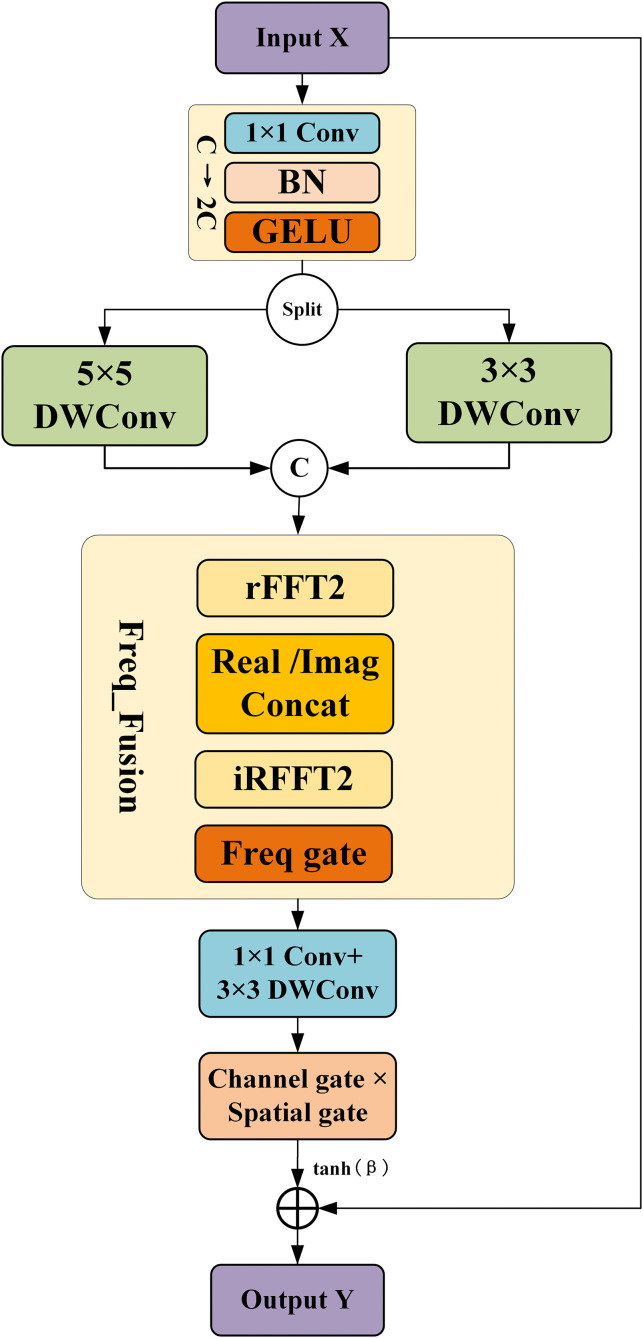
Structure of the FFCM.


As shown in Fig 3, FFCM contains three main stages: local multi-scale spatial extraction, frequency-domain gated fusion, and channel-spatial residual refinement. Given an input feature *X*∈$R^{C\times H\times W}$, FFCM first expands the channel dimension by a 1 × 1 Conv-BN-GELU block and then splits the expanded feature into two channel groups:

$$\begin{array}{c} Z=\phi (BN({Conv}_{\text{1}\times \text{1}}(X))),\left[Z_{\text{1}},Z_{\text{2}}\right]=Split(Z) \end{array}$$ (1)

where $\left(Z\in R^{\text{2}C\times H\times W}),(Z_{\text{1}},Z_{\text{2}}\in R^{C\times H\times W}\right)$, and ϕ(·) denotes the GELU activation. The two feature groups are processed by 3 × 3 and 5 × 5 depthwise convolution branches, respectively:


$$\begin{array}{c} L_{\text{3}}=\phi \left(\text{BN}\left({\text{DWConv}}_{\text{3}\times \text{3}}(Z_{\text{1}})\right)\right) \end{array}$$ (2)

$$\begin{array}{c} L_{\text{5}}=\phi \left(\text{BN}\left({\text{DWConv}}_{\text{5}\times \text{5}}(Z_{\text{2}})\right)\right) \end{array}$$ (3)

$$\begin{array}{c} L=\text{Concat}(L_{\text{3}},L_{\text{5}}) \end{array}$$ (4)

The 3 × 3 branch captures fine local crack patterns, while the 5 × 5 branch provides a slightly larger local receptive field. The concatenated feature *L* is then fed into the Freq_Fusion block for frequency-domain interaction.

Inside the Freq_Fusion block, *L* is first divided into two channel groups and projected by lightweight 1 × 1 Conv-BN-GELU transformations to obtain a local reference feature:


Freq_Fusion (5)

$$\begin{array}{c} L_{\text{0}}=\text{Concat}\left(\psi_{\text{1}}(L_{\text{1}}),\psi_{\text{2}}(L_{\text{2}})\right) \end{array}$$ (6)

where $\psi_{\text{1}}\left(\cdot \right)$and $\psi_{\text{2}}\left(\cdot \right)$denote 1 × 1 Conv-BN-GELU transformations. A real-valued two-dimensional fast Fourier transform is then applied to ${\text{L}}_{\text{0}}$:


$$\begin{array}{c} \hat{L}=F_{r}(L_{\text{0}}) \end{array}$$ (7)

The real and imaginary components of $\hat{L}$ are concatenated and mixed by a 1 × 1 convolution:


$$\begin{array}{c} Q=\phi \left(BN(Conv_{\text{1}\times \text{1}}(Concat(Re(\hat{L}),Im(\hat{L}))))\right) \end{array}$$ (8)

After splitting *Q* into real and imaginary parts, the inverse real-valued FFT reconstructs the spatial-domain Fourier-branch feature:


$$\begin{array}{c} F_{\text{freq}}=F_{r}^{-\text{1}}(Q_{r}+jQ_{i}) \end{array}$$ (9)

To regulate the contribution of the reconstructed branch, a lightweight frequency-branch channel gate is applied after the inverse Fourier transform:


$$\begin{array}{c} G_{f}=\sigma \left({\text{Conv}}_{\text{1}\times \text{1}}^{\left(\text{2}\right)}\left(\phi \left({\text{Conv}}_{\text{1}\times \text{1}}^{\left(\text{1}\right)}\left(\text{GAP}(F_{\text{freq}})\right)\right)\right)\right) \end{array}$$ (10)

$$\begin{array}{c} Y_{f}=\phi \left(\text{BN}\left(L_{\text{0}}+\text{tanh}(\gamma )\left(F_{\text{freq}}⊙G_{f}\right)\right)\right) \end{array}$$ (11)

where *G*_*f*_ is the frequency-branch channel gate, γ is a learnable residual scaling parameter, σ(·) denotes the Sigmoid function, and ⊙ denotes element-wise multiplication. The bounded term tanh(γ) controls the contribution of the reconstructed Fourier branch and improves training stability. Because the gate is applied after the inverse transform, it reweights reconstructed feature channels rather than selecting individual frequency bins.

After gated Fourier-branch fusion, the feature is projected back to *C* channels using a 1 × 1 convolution followed by a 3 × 3 depthwise convolution:


$$\begin{array}{c} U=\phi \left(\text{BN}\left({\text{DWConv}}_{\text{3}\times \text{3}}\left(\phi \left(\text{BN}\left({\text{Conv}}_{\text{1}\times \text{1}}(Y_{f})\right)\right)\right)\right)\right) \end{array}$$ (12)

Finally, channel and spatial gates are jointly applied to adaptively reweight the projected feature before residual fusion:


$$\begin{array}{c} G_{c}(U)=\sigma \left({\text{Conv}}_{\text{1}\times \text{1}}^{\left(\text{2}\right)}\left(\phi \left({\text{Conv}}_{\text{1}\times \text{1}}^{\left(\text{1}\right)}\left(\text{GAP}(U)\right)\right)\right)\right) \end{array}$$ (13)

$$\begin{array}{c} G_{s}(U)=\sigma \left({\text{Conv}}_{\text{7}\times \text{7}}(U)\right) \end{array}$$ (14)

$$\begin{array}{c} Y_{\text{FFCM}}=X+\text{tanh}(\beta )\left(U⊙G_{c}(U)⊙G_{s}(U)\right) \end{array}$$ (15)

where $G_{c}\left(U\right)$ and $G_{s}\left(U\right)$ denote the channel gate and spatial gate, respectively, and β is another learnable residual scaling parameter. Therefore, FFCM does not replace the original feature representation. Instead, it uses a bounded residual path to refine the input feature with local multi-scale information and full-spectrum Fourier-domain component interaction. The module does not assume that high-frequency components are exclusively associated with cracks, because crack structures and background textures may coexist in similar spectral regions. Accordingly, FFCM is interpreted as an auxiliary gated Fourier-domain refinement component rather than a module that explicitly amplifies predefined frequency bands.

### 3.2. Residual edge enhancement module

The Residual Edge Enhancement Module (REEM) is designed to enhance crack boundary features in the detection head. Bridge cracks are usually slender, irregular, and discontinuous, and their edge responses can be weakened by repeated convolution and downsampling. In addition, concrete texture, stains, corrosion, and shadows may produce crack-like responses, which increases the difficulty of distinguishing true cracks from background interference. To address these problems, REEM introduces five complementary edge-related branches, including a local depthwise branch, a dilated depthwise branch, a horizontal stripe branch, a vertical stripe branch, and a Sobel-prior branch. The extracted edge features are fused and regulated by channel and spatial gates, and the final edge-enhanced feature is added back to the input through a bounded residual connection.

**Fig 4:**
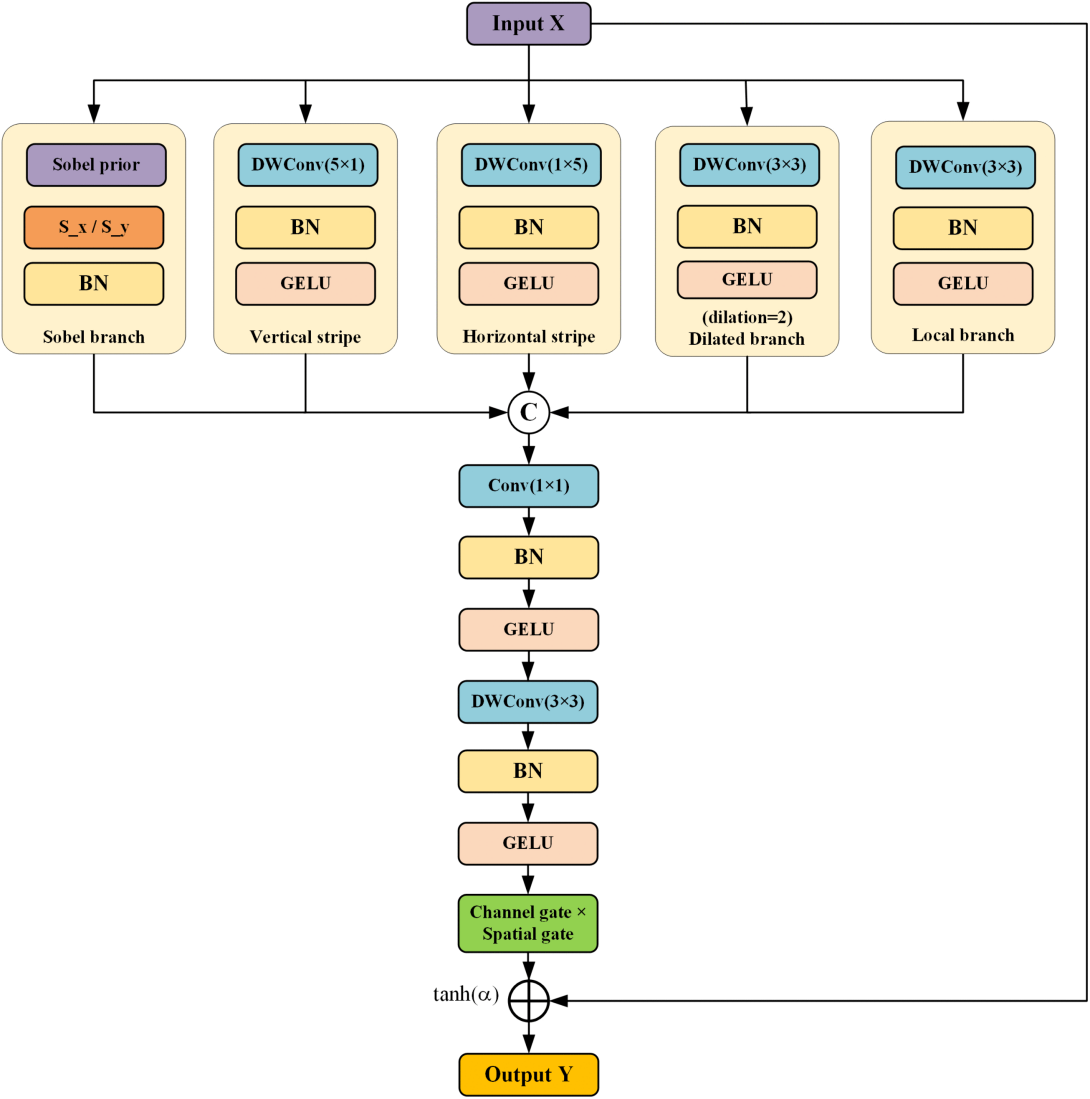
Structure of the REEM.


As shown in Fig 4, given an input feature *X*∈${\text{R}}^{C\times H\times W}$, the local depthwise branch uses a 3 × 3 depthwise convolution to capture fine local crack boundaries:

$$\begin{array}{c} E_{l}=\phi \left(\text{BN}\left({\text{DWConv}}_{\text{3}\times \text{3}}(X)\right)\right) \end{array}$$ (16)

where $E_{l}$ denotes the local edge feature, ${\text{DWConv}}_{\text{3}\times \text{3}}$ denotes a 3 × 3 depthwise convolution, BN(·) denotes batch normalization, and ϕ(·) denotes the GELU activation function.

To enlarge the local receptive field without greatly increasing computational cost, the dilated branch uses a 3 × 3 depthwise convolution with dilation rate d=2:


$$\begin{array}{c} E_{d}=\phi \left(\text{BN}\left({\text{DWConv}}_{\text{3}\times \text{3},d=\text{2}}(X)\right)\right) \end{array}$$ (17)

where $E_{d}$ denotes the dilated edge feature. This branch helps capture weak and discontinuous crack responses over a slightly larger neighborhood.

The horizontal and vertical stripe branches are introduced to model elongated crack structures. The horizontal stripe branch uses a 1 × 5 depthwise convolution:


$$\begin{array}{c} E_{h}=\phi \left(\text{BN}\left({\text{DWConv}}_{\text{1}\times \text{5}}(X)\right)\right) \end{array}$$ (18)

and the vertical stripe branch uses a 5 × 1 depthwise convolution:


$$\begin{array}{c} E_{v}=\phi \left(\text{BN}\left({\text{DWConv}}_{\text{5}\times \text{1}}(X)\right)\right) \end{array}$$ (19)

Here, $E_{h}$ and $E_{v}$ represent the horizontal and vertical stripe features, respectively. Although cracks can be oblique or curved, the horizontal and vertical stripe branches provide two directional bases for enhancing elongated structures. When combined with the local, dilated, and Sobel-prior branches, they help improve the continuity of responses along slender crack regions.

In addition to learnable convolutional branches, REEM introduces a fixed Sobel-gradient prior to provide explicit edge guidance. The Sobel branch computes the gradient magnitude using fixed horizontal and vertical Sobel kernels:


$$\begin{array}{c} E_{s}=\text{BN}\left(\sqrt{(S_{x}*X{)}^{\text{2}}+(S_{y}*X{)}^{\text{2}}+\epsilon }\right) \end{array}$$ (20)

Where S_*x*_ and S_y_ denote the fixed horizontal and vertical Sobel kernels, respectively, * denotes channel-wise convolution, ϵ is a small constant for numerical stability, and ${\text{E}}_{\text{s}}$ is the Sobel-prior edge feature. This branch provides a non-learnable gradient cue that complements the learnable convolutional edge features.

The five branch outputs are concatenated along the channel dimension:


$$\begin{array}{c} E_{\text{0}}=\text{Concat}(E_{l},E_{d},E_{h},E_{v},E_{s}) \end{array}$$ (21)

After concatenation, a 1 × 1 convolution is used to fuse the multi-branch features and reduce the channel dimension. A subsequent 3 × 3 depthwise convolution further refines the fused edge feature:


$$\begin{array}{c} E_{f}=\phi \left(\text{BN}\left({\text{DWConv}}_{\text{3}\times \text{3}}\left(\phi \left(\text{BN}\left({\text{Conv}}_{\text{1}\times \text{1}}(E_{\text{0}})\right)\right)\right)\right)\right) \end{array}$$ (22)

where *E*_*f*_ denotes the fused edge feature. The 1 × 1 convolution performs cross-branch channel fusion, while the 3 × 3 depthwise convolution further strengthens local spatial consistency with low computational overhead.

To suppress irrelevant background responses and emphasize crack-related features, channel and spatial gates are applied to the fused edge feature. The channel gate is defined as:


$$\begin{array}{c} G_{c}(E_{f})=\sigma \left({\text{Conv}}_{\text{1}\times \text{1}}^{\left(\text{2}\right)}\left(\phi \left({\text{Conv}}_{\text{1}\times \text{1}}^{\left(\text{1}\right)}\left(\text{GAP}(E_{f})\right)\right)\right)\right) \end{array}$$ (23)

and the spatial gate is defined as:


$$\begin{array}{c} G_{s}(E_{f})=\sigma \left({\text{Conv}}_{\text{7}\times \text{7}}(E_{f})\right) \end{array}$$ (24)

where $G_{c}(E_{f})$ and $G_{s}(E_{f})$ denote the channel and spatial gates, respectively, GAP(·) denotes global average pooling, and σ(·) is the Sigmoid activation function. The gated edge feature is obtained as:


$$\begin{array}{c} E_{g}=E_{f}⊙G_{c}(E_{f})⊙G_{s}(E_{f}) \end{array}$$ (25)

Where ⊙ denotes element-wise multiplication.

Finally, REEM adopts a bounded residual connection to preserve the original feature representation while adaptively enhancing crack edges:


$$\begin{array}{c} Y_{\text{REEM}}=X+\text{tanh}(\alpha )E_{g} \end{array}$$ (26)

where α is a learnable residual scaling parameter. The bounded term tanh(α) controls the enhancement amplitude of the edge feature and helps avoid excessive amplification of background texture. Through this residual design, REEM enhances crack boundary information while maintaining the stability of the original YOLO11 feature representation.

Overall, REEM integrates learnable local, dilated, and directional stripe convolutions with a fixed Sobel-gradient prior. The local branch captures fine boundary details, the dilated branch enlarges the local receptive field, the horizontal and vertical stripe branches enhance elongated crack-like structures, and the Sobel branch provides explicit gradient guidance. The subsequent channel-spatial gating and bounded residual connection allow REEM to improve crack-edge representation while reducing the risk of enhancing irrelevant concrete texture.

## 4. Experimental setup

### 4.1. Experimental datasets

Experiments were conducted using two public data sources: GYU-DET and Crack500. For the main experiments, we extracted the crack-category subset from the GYU-DET bridge surface defect dataset [35] and refer to this subset as GYU-DET-Crack in this paper. GYU-DET-Crack was used for module ablation, comparative experiments, repeated-seed stability analysis, and visualization analysis. The original train, validation, and test partitions of GYU-DET were retained during subset construction. For each partition, only annotations belonging to the crack category were retained and remapped to a single detection class. Images containing at least one crack annotation were kept as positive samples, and a subset of non-crack images was retained as hard negative samples to evaluate false-positive responses on background bridge-surface images. The number of retained negative images in each partition was limited to approximately half of the number of positive images. The resulting GYU-DET-Crack dataset contained 2440 training images, including 813 background images; 219 validation images, including 73 background images; and 225 test images, including 75 background images and 404 annotated crack instances. No offline augmentation-based dataset expansion or generation of transformed image copies was performed during subset construction. All model training and evaluation procedures on GYU-DET-Crack used this fixed split.

Crack500 [36] was used as an additional public benchmark to examine whether the modified architecture remained effective after independent retraining on a different data source. Crack500 provides pixel-level crack annotations. Since this study focuses on object detection, the crack masks were converted into bounding-box annotations by extracting connected crack regions and generating their minimum enclosing rectangles. The original cropped train/validation/test split of Crack500 was retained, including 1,896 training images, 348 validation images, and 1124 test images. The converted validation and test sets contained 582 and 1990 crack instances, respectively. No offline augmentation-based dataset expansion was applied to Crack500. Crack500 was used only for additional-dataset validation, whereas all ablation, comparative, repeated-seed, and visualization experiments were conducted on GYU-DET-Crack.

### 4.2. Experimental environment

All experiments were conducted on a Windows 10 64-bit platform equipped with an Intel Core i5-12600KF CPU and an NVIDIA GeForce RTX 4060 Ti GPU with 16 GB memory. The software environment included Python 3.9.25, PyTorch 2.5.1 with CUDA 12.1 support, NVIDIA driver 560.94, and Ultralytics 8.3.243 [19]. All models were trained for 100 epochs with an input size of 640 × 640 and a batch size of 16. The optimizer was automatically selected by Ultralytics, and AdamW was used during training. For the repeated experiments, seed 0, seed 1, and seed 2 used the same dataset split, training epochs, image size, batch size, optimizer setting, and evaluation protocol. For the Crack500 experiment, YOLO11n and YOLO11-FR were trained and evaluated using the same image size, training epochs, batch size, seed, optimizer setting, and evaluation protocol as the main experiments.

### 4.3. Evaluation metrics

Five evaluation metrics were used to assess crack detection performance: Precision (P), Recall (R), F1-score, mAP50, and mAP50-95. Precision measures the proportion of correctly detected crack samples among all samples predicted as cracks and is defined as:


$$\begin{array}{c} P=\frac{TP}{TP+FP} \end{array}$$ (27)

Recall measures the proportion of correctly detected crack samples among all actual crack samples and is defined as:


$$\begin{array}{c} R=\frac{TP}{TP+FN} \end{array}$$ (28)

The F1-score is the harmonic mean of Precision and Recall and is defined as:


$$\begin{array}{c} F\text{1}=\frac{\text{2}\times P\times R}{P+R} \end{array}$$ (29)

Average Precision (AP) is calculated as the area under the precision-recall curve:


$$\begin{array}{c} AP=\int_{\text{0}}^{\text{1}}P(r),dr \end{array}$$ (30)

where *P*(*r*) denotes precision as a function of recall (r). Since this study detects a single crack category, mAP is equivalent to the AP of the crack class. mAP50 denotes AP at an Intersection over Union (IoU) threshold of 0.50:


$$\begin{array}{c} mAP_{\text{5}\text{0}}=AP_{\text{0}.\text{5}\text{0}} \end{array}$$ (31)

mAP50-95 denotes the mean AP over IoU thresholds from 0.50 to 0.95 with an interval of 0.05:


$$\begin{array}{c} mAP_{\text{5}\text{0}-\text{9}\text{5}}=\frac{\text{1}}{\text{1}\text{0}}\sum_{i=\text{0}}^{\text{9}}AP_{\text{0}.\text{5}\text{0}+\text{0}.\text{0}\text{5}i} \end{array}$$ (32)

In these formulas, TP denotes true positives, FP denotes false positives, and FN denotes false negatives. These metrics evaluate detection accuracy, detection completeness, and localization quality under different IoU thresholds.

## 5. Experimental results

### 5.1. Ablation experiment

#### 5.1.1. Ablation of FFCM and REEM.

To evaluate the individual and combined effects of FFCM and REEM, an ablation experiment was conducted on the GYU-DET-Crack test set. As shown in Table 2, FFCM and REEM contribute to YOLO11-FR in different ways. When FFCM is introduced alone, recall increases from 38.1% to 42.1%, and F1-score increases from 41.3% to 43.3%. Meanwhile, mAP50-95 increases from 18.4% to 18.8%, although precision and mAP50 show slight decreases. This indicates that FFCM improves the detection sensitivity of YOLO11n and provides a certain localization benefit under stricter IoU evaluation, but its effect is not uniformly reflected in all metrics when used as a single module.

**Table 2:** Ablation results of FFCM and REEM on the GYU-DET-Crack test set.

Algorithm	Precision/%	Recall/%	F1/%	mAP50/%	mAP50-95/%	Params/M	GFLOPs	FPS
YOLO11n	45.2	38.1	41.3	38.2	18.4	2.58	6.3	303.0
YOLO11n + FFCM	44.6	42.1	43.3	38.0	18.8	4.15	7.6	277.8
YOLO11n + REEM	48.2	44.1	46.1	41.5	21.6	3.10	7.3	238.1
YOLO11-FR	45.3	42.8	44.0	41.6	22.1	4.67	8.6	208.3

Compared with FFCM, REEM provides a stronger single-module improvement. YOLO11n + REEM improves precision by 3.0 percentage points, recall by 6.0 percentage points, F1-score by 4.8 percentage points, mAP50 by 3.3 percentage points, and mAP50-95 by 3.2 percentage points compared with YOLO11n. These results show that residual edge enhancement is highly effective for bridge crack detection, where boundary continuity and edge responses are critical for accurate localization.

When FFCM and REEM are combined, YOLO11-FR achieves the best mAP50 and mAP50-95 among the four variants. Compared with YOLO11n + REEM, YOLO11-FR further improves mAP50-95 from 21.6% to 22.1%, indicating that the Fourier-domain refinement introduced by FFCM provides complementary localization-oriented information to the edge-enhanced features generated by REEM. Although precision, recall, and F1-score are slightly lower than those of YOLO11n + REEM, the complete model obtains the highest AP-based detection accuracy, especially under stricter localization criteria.

In terms of computational efficiency, the number of parameters increases from 2.58 M to 4.67 M, GFLOPs increase from 6.3 to 8.6, and FPS decreases from 303.0 to 208.3. The additional computational cost is mainly introduced by the two feature enhancement modules, but YOLO11-FR still maintains real-time inference capability on the tested hardware platform.

**Fig 5:**
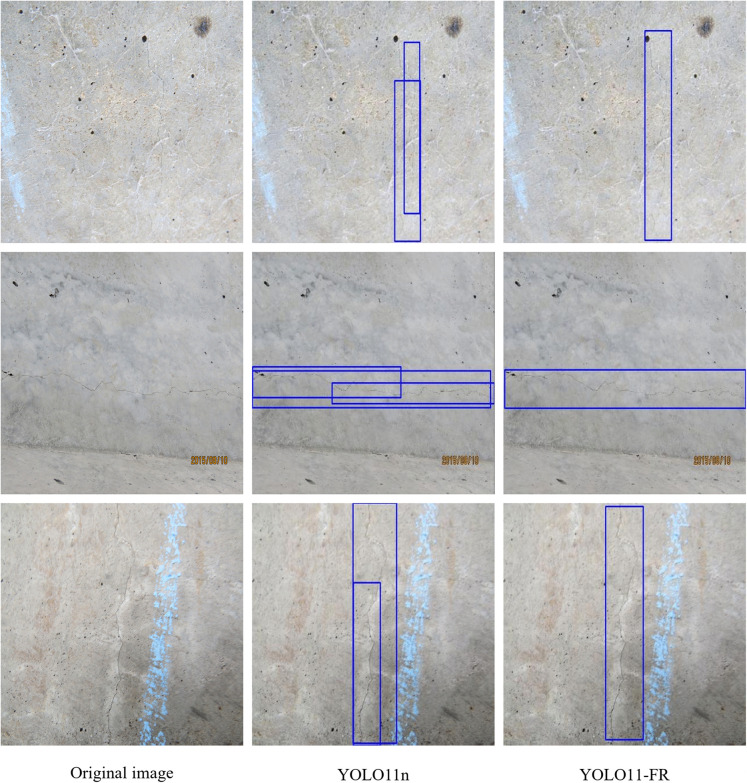
Comparison of detection results in the ablation experiment. Source images are from the GYU-DET dataset [35], Science Data Bank, licensed under CC BY 4.0.

Fig 5 shows representative detection results of YOLO11n and YOLO11-FR on the GYU-DET-Crack test set. Compared with YOLO11n, YOLO11-FR produces more complete and less fragmented localization results for several slender cracks under non-uniform concrete backgrounds with texture noise, surface stains, and low crack contrast. In particular, YOLO11n tends to generate multiple overlapping or discontinuous bounding boxes for long crack regions, whereas YOLO11-FR provides more continuous crack-region coverage. These qualitative results provide visual support for the quantitative improvements reported above.

#### 5.1.2. Ablation of stripe-convolution branches in REEM.

To verify the contribution of the horizontal and vertical stripe-convolution branches in REEM, an additional variant, YOLO11 + REEM without stripe branches, was constructed by removing the 1 × 5 and 5 × 1 depthwise branches. The remaining components, including the local 3 × 3 branch, dilated 3 × 3 branch, Sobel-gradient prior, channel and spatial gates, and bounded residual scaling, were retained.

As shown in Table 3, removing the stripe-convolution branches slightly increases precision from 48.2% to 48.7%, but reduces recall from 44.1% to 38.6%, F1-score from 46.1% to 43.0%, mAP50 from 41.5% to 38.7%, and mAP50-95 from 21.6% to 19.8%. This indicates that the model without stripe-convolution branches becomes more conservative in crack prediction but misses more crack instances and suffers from reduced overall localization quality. Therefore, the horizontal and vertical stripe-convolution branches are beneficial for improving detection completeness and enhancing directional responses along elongated crack structures.

**Table 3:** Ablation results of stripe-convolution branches in REEM.

Algorithm	Stripe branches	Precision/%	Recall/%	F1/%	mAP50/%	mAP50-95/%
YOLO11 + REEM w/o stripe	No	48.7	38.6	43.0	38.7	19.8
YOLO11 + REEM	Yes	48.2	44.1	46.1	41.5	21.6

### 5.2. Comparative performance and computational efficiency analysis

To further evaluate the proposed method, YOLO11-FR was compared with Faster R-CNN, YOLOv10n, YOLO11n, and BD-YOLOv8s. YOLOv10n and YOLO11n were used as lightweight YOLO-family baselines, and Faster R-CNN was included as a representative two-stage detector. In response to the need for a more task-specific comparison, BD-YOLOv8s was included as a bridge-defect-specific detector, providing a more targeted comparison than generic detection baselines alone. Table 4 summarizes both the detection performance and computational efficiency of the compared methods.

**Table 4:** Comparison of detection performance and computational efficiency of different models.

Algorithm	Precision/%	Recall/%	F1/%	mAP50/%	mAP50-95/%	Params/M	GFLOPs	FPS
Faster R-CNN	40.2	47.5	43.5	38.1	17.8	41.3	134.0*	22.0
YOLOv10n	42.9	35.5	38.8	34.1	17.5	2.3	6.5	303.0
YOLO11n	45.2	38.1	41.3	38.2	18.4	2.6	6.3	303.0
BD-YOLOv8s	44.9	42.3	43.6	39.4	18.6	11.4	27.8	100.0
YOLO11-FR	45.3	42.8	44.0	41.6	22.1	4.7	8.6	208.3

* The GFLOPs of Faster R-CNN were estimated using fvcore. Since Faster R-CNN contains dynamic proposal and ROI operations, the reported GFLOPs are approximate.

YOLO11-FR achieves the highest mAP50 and mAP50-95 among the compared detectors, reaching 41.6% and 22.1%, respectively. Compared with the YOLO11n baseline, YOLO11-FR improves mAP50 from 38.2% to 41.6% and mAP50-95 from 18.4% to 22.1%. Although the additional FFCM and REEM modules increase the parameter count and computational cost, the proposed model still achieves 208.3 FPS on the experimental platform, indicating that it maintains real-time inference capability. Compared with BD-YOLOv8s, YOLO11-FR improves mAP50 by 2.2 percentage points and mAP50-95 by 3.5 percentage points, while reducing the parameter count from 11.35M to 4.67M and the computational cost from 27.8 GFLOPs to 8.6 GFLOPs. Its inference speed is also more than twice that of BD-YOLOv8s. Faster R-CNN achieves the highest recall of 47.5%; however, it has 41.3M parameters and approximately 134.0 GFLOPs, and its inference speed is only 22.0 FPS. This limits its suitability for real-time bridge crack screening compared with lightweight YOLO-based detectors. Overall, YOLO11-FR provides a favorable balance between detection accuracy and computational efficiency.

**Fig 6:**
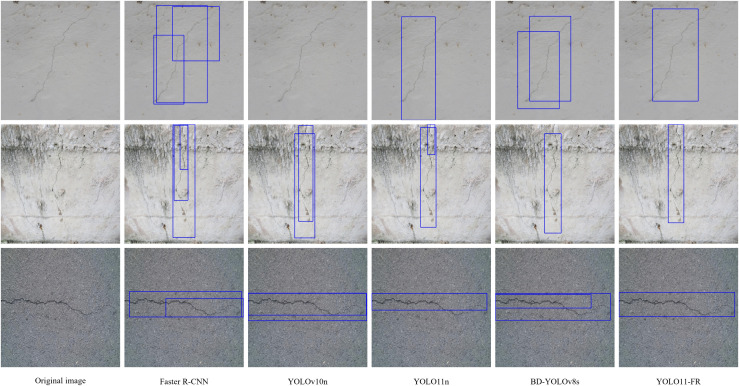
Visual comparison of detection results produced by different detectors on representative GYU-DET-Crack test images. Source images are from the GYU-DET dataset [35], Science Data Bank, licensed under CC BY 4.0.

Fig 6 shows representative detection results of different algorithms on the GYU-DET-Crack test set. In the selected examples, YOLO11-FR generally provides more continuous and stable crack localization results for slender, low-contrast, and bridge-surface crack cases. Compared with the baseline detectors, YOLO11-FR tends to reduce missed detections and improve the localization continuity of crack regions. These visual comparisons provide qualitative support for the quantitative results reported in Table 4.

### 5.3. Repeated-seed stability analysis

To reduce the influence of random initialization and data-loading order, YOLO11n and YOLO11-FR were trained and evaluated using three random seeds under identical configurations. Table 5 reports the individual results for seed 0, seed 1, and seed 2, together with the corresponding mean ± standard deviation. Compared with YOLO11n, YOLO11-FR improves the mean precision from 41.9% to 47.0%, recall from 40.8% to 43.6%, F1-score from 41.2% to 45.2%, mAP50 from 36.7% to 41.3%, and mAP50-95 from 17.5% to 20.8%. The corresponding improvements are 5.1, 2.8, 4.0, 4.6, and 3.3 percentage points, respectively.

**Table 5:** Repeated-seed results of YOLO11n and YOLO11-FR.

Algorithm	Seed	Precision/%	Recall/%	F1/%	mAP50/%	mAP50-95/%
YOLO11n	0	45.2	38.1	41.3	38.2	18.4
YOLO11n	1	43.3	42.1	42.7	38.5	18.3
YOLO11n	2	37.2	42.1	39.5	33.5	15.8
YOLO11n	Mean ± SD	41.9 ± 4.2	40.8 ± 2.3	41.2 ± 1.6	36.7 ± 2.8	17.5 ± 1.5
YOLO11-FR	0	45.3	42.8	44.0	41.6	22.1
YOLO11-FR	1	49.1	42.8	45.7	41.5	20.1
YOLO11-FR	2	46.7	45.3	46.0	40.8	20.2
YOLO11-FR	Mean ± SD	47.0 ± 1.9	43.6 ± 1.4	45.2 ± 1.1	41.3 ± 0.4	20.8 ± 1.1

In addition, YOLO11-FR obtains higher mAP50 and mAP50-95 than YOLO11n under all three random seeds. The standard deviations of YOLO11-FR are also lower across all reported metrics, especially for mAP50, where the standard deviation decreases from 2.8 to 0.4. These results indicate that YOLO11-FR provides more stable detection performance than YOLO11n under different random seeds, while the repeated-seed experiment is used as supporting evidence for robustness rather than a formal statistical significance test.

### 5.4. Eigen-CAM visualization analysis

**Fig 7:**
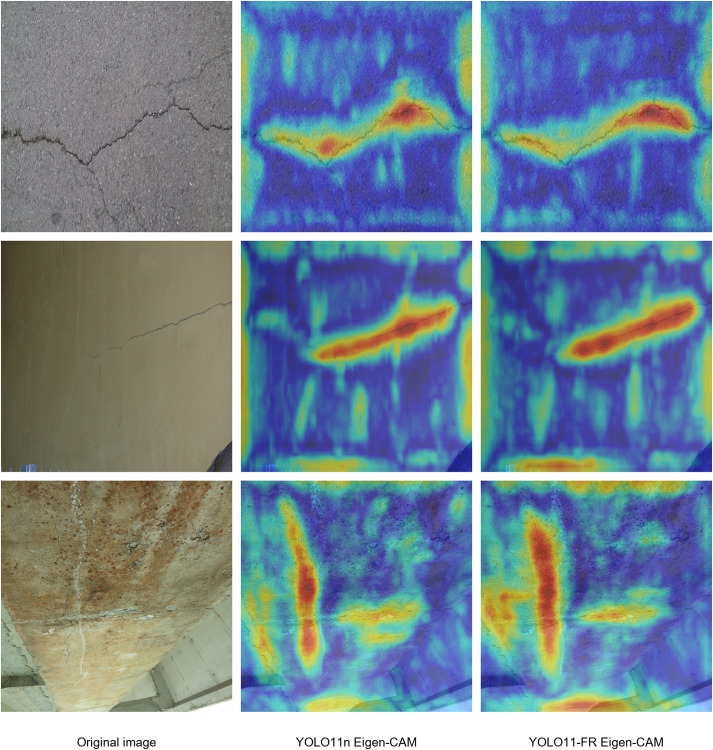
Eigen-CAM visualization comparison between YOLO11n and YOLO11-FR on representative GYU-DET-Crack test images. Source images are from the GYU-DET dataset [35], Science Data Bank, licensed under CC BY 4.0.

To further examine the feature-activation behavior of the models, Eigen-CAM [37] was used to visualize the activation regions of YOLO11n and YOLO11-FR on representative GYU-DET-Crack test images, as shown in Fig 7. Each row contains the original image, the Eigen-CAM result of YOLO11n, and the Eigen-CAM result of YOLO11-FR. Compared with YOLO11n, YOLO11-FR generally produces more continuous and concentrated activation responses around crack regions, especially for slender, irregular, and low-contrast cracks under non-uniform concrete backgrounds. Although some background responses remain, the activation regions of YOLO11-FR are more consistently aligned with crack-relevant areas in the selected examples. This qualitative pattern is consistent with the intended effect of high-level Fourier-domain refinement and multi-scale edge enhancement. These visualizations provide supplementary evidence for the improved crack-region attention of YOLO11-FR.

### 5.5. Additional benchmark evaluation on Crack500

To further evaluate the effectiveness of YOLO11-FR on an additional public crack benchmark, YOLO11n and YOLO11-FR were independently trained and evaluated on the converted Crack500 detection subset. The official cropped train/validation/test split was retained, and the pixel-level crack masks were converted into bounding-box annotations to match the object-detection setting of this study. The training set contained 1,896 images, the validation set contained 348 images, and the test set contained 1,124 images with 1,990 crack instances. Because both models were retrained on Crack500, this experiment evaluates additional-benchmark effectiveness after retraining rather than zero-shot cross-dataset transfer.

As shown in Table 6, YOLO11-FR achieves higher detection performance than YOLO11n on the Crack500 test set. Compared with YOLO11n, YOLO11-FR slightly increases precision from 69.5% to 69.8%, recall from 52.3% to 53.6%, F1-score from 59.7% to 60.6%, mAP50 from 56.0% to 57.7%, and mAP50-95 from 32.7% to 34.8%. The improvement in mAP50-95 indicates that YOLO11-FR provides better localization performance under stricter IoU thresholds. These results support the effectiveness of the complete YOLO11-FR architecture on a second public crack benchmark after independent retraining.

**Table 6:** Additional benchmark results on the converted Crack500 dataset.

Algorithm	Precision/%	Recall/%	F1/%	mAP50/%	mAP50-95/%
YOLO11n	69.5	52.3	59.7	56.0	32.7
YOLO11-FR	69.8	53.6	60.6	57.7	34.8

## 6. Discussion and limitations

The experimental results show that YOLO11-FR improves crack detection performance over YOLO11n on GYU-DET-Crack. The ablation results indicate that the two proposed modules contribute to the model in different ways. REEM provides the main and more consistent improvement across precision, recall, F1-score, mAP50, and mAP50-95, highlighting the importance of crack-oriented edge refinement for slender and low-contrast cracks. FFCM plays a complementary high-level refinement role: although FFCM alone mainly improves recall, F1-score, and mAP50-95, combining FFCM with REEM further improves the AP-based localization performance of the complete YOLO11-FR model. The Eigen-CAM visualization further suggests that YOLO11-FR can generate more continuous and crack-relevant activation responses in representative test images, providing qualitative support for the quantitative results.

The additional Crack500 experiment further supports the effectiveness of YOLO11-FR after independent retraining on another public crack benchmark. Compared with YOLO11n, YOLO11-FR improves mAP50 from 56.0% to 57.7% and mAP50-95 from 32.7% to 34.8% on the converted Crack500 test set. These results indicate that the proposed architecture can maintain its effectiveness on an additional crack dataset after retraining, suggesting its potential applicability beyond the main GYU-DET-Crack dataset.

Several limitations remain. First, although potential data leakage caused by offline augmentation-based dataset expansion was avoided by using an image-level split and by not generating transformed image copies across subsets, the main experiments were conducted on the crack subset extracted from GYU-DET, a public bridge surface defect dataset. This dataset cannot fully represent all bridge types, material conditions, acquisition devices, geographic regions, and inspection environments. Crack500 was introduced as an additional public crack benchmark, but it is not a bridge-specific inspection dataset. Therefore, further validation on more bridge-oriented datasets and real inspection scenarios is still needed.

Second, real bridge inspection may involve UAV viewpoints, wet surfaces, corrosion, occlusion, strong shadows, low illumination, and motion blur, which are not exhaustively covered in the current experiments. In addition, this study focuses on bounding-box-based crack detection rather than pixel-level segmentation, so YOLO11-FR does not directly estimate crack width, crack length, or pixel-level crack area.

## 7. Conclusion

This study proposed YOLO11-FR for bridge crack detection by integrating frequency-domain feature interaction and crack-oriented edge refinement into the YOLO11 detection head. The proposed FFCM provides gated full-spectrum Fourier-domain refinement on the high-level P5 feature, while REEM enhances multi-scale crack boundary representation through local, dilated, directional stripe, and Sobel-prior branches. Both modules are inserted through bounded residual pathways to preserve the original YOLO11 feature-fusion route and improve crack localization while retaining real-time inference capability.

Experiments on GYU-DET-Crack show that YOLO11-FR improves mAP50 from 38.2% to 41.6% and mAP50-95 from 18.4% to 22.1% compared with YOLO11n. The repeated-seed analysis and additional Crack500 experiment further support the effectiveness and stability of the proposed architecture. Compared with BD-YOLOv8s, YOLO11-FR also achieves higher AP-based accuracy with fewer parameters and lower computational cost. These results suggest that YOLO11-FR provides a crack-oriented detection approach for rapid bridge crack screening under complex concrete surface backgrounds.

Future work will focus on validating YOLO11-FR on more bridge-specific datasets and real UAV-based bridge inspection scenes under adverse surface and imaging conditions. In addition, extending the current bounding-box detection framework toward pixel-level crack segmentation and quantitative crack measurement will be explored to support more detailed bridge condition assessment.
